# Discussion on the Rehabilitation of Stroke Hemiplegia Based on Interdisciplinary Combination of Medicine and Engineering

**DOI:** 10.1155/2021/6631835

**Published:** 2021-03-17

**Authors:** Xiaowei Sun, Ke Xu, Yuqing Shi, Hongtao Li, Ruobing Li, Siyu Yang, Hong Jin, Chuwen Feng, Baitao Li, Chunyue Xing, Yuanyuan Qu, Qingyong Wang, Yinghua Chen, Tiansong Yang

**Affiliations:** ^1^Heilongjiang University of Chinese Medicine, 24 Heping Road, Xiangfang District, Harbin 8615-0040, China; ^2^First Affiliated Hospital, Heilongjiang University of Chinese Medicine, 26 Heping Road, Xiangfang District, Harbin 8615-0040, China; ^3^Shenzhen People's Hospital, Second Clinical Medical College of Jinan University, Department of Rehabilitation Medicine, Shenzhen 518120, China

## Abstract

Interdisciplinary combinations of medicine and engineering are part of the strategic plan of many universities aiming to be world-class institutions. One area in which these interactions have been prominent is rehabilitation of stroke hemiplegia. This article reviews advances in the last five years of stroke hemiplegia rehabilitation via interdisciplinary combination of medicine and engineering. Examples of these technologies include VR, RT, mHealth, BCI, tDCS, rTMS, and TCM rehabilitation. In this article, we will summarize the latest research in these areas and discuss the advantages and disadvantages of each to examine the frontiers of interdisciplinary medicine and engineering advances.

## 1. Introduction

**Figure 1:**
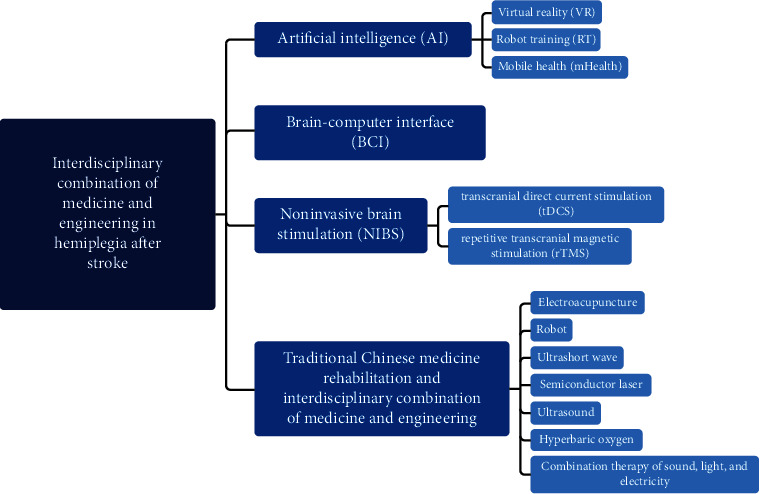
Application of interdisciplinary medicine and engineering approaches and technologies in the field of stroke hemiplegia rehabilitation.

Stroke is a serious cerebrovascular disease characterized by sudden and acute onset and rapid neurological deficits, which is the world's leading cause of disability and the second leading cause of death [1], leaving 80% of patients having varying degrees of lifetime neurological deficits [2]. As the global aging problem is getting worse, the positive correlation between stroke and age means that the incidence of stroke will only continue to rise. Stroke incidence is also trending toward even younger patients due to factors such as irregular work life and infrequent rest, a growing sense of pressure and anxiety, poor eating habits, and many other reasons. Hemiplegia is one of the most common symptoms of stroke and significantly affects the patient's quality of life by reducing their ability to perform activities of daily living. While the rehabilitation of hemiplegic stroke patients has commanded considerable attention in society and medicine, a severe shortage of rehabilitation therapists leads to inconsistent traditional rehabilitation training results. Thus, new treatments borne out of interdisciplinary medicine and engineering methods offer the potential to provide superior care for hemiplegic stroke patients. Such methods can not only promote the recovery of the patient by stimulating nerve remodeling, but also reduce physician workload. As shown in Figure 1, we will describe advances in the interdisciplinary combination of medicine and engineering for stroke hemiplegia rehabilitation through four primary domains: artificial intelligence, brain-computer interface, noninvasive brain stimulation, and traditional Chinese medicine.

## 2. Artificial Intelligence (AI)

### 2.1. Virtual Reality (VR)

Virtual reality (VR) combines the VR technology characteristics of autonomy, interactivity, and presence with rehabilitation training, to provide novel methods for stroke patients to undergo neurorehabilitation in virtual environments [3]. VR is either (1) immersive, in which participants act within a computer-generated simulation world, often as an avatar, or (2) nonimmersive VR, which uses an environment that includes a 3D graphics game system. Users can use a keyboard, mouse, or other game interface devices to interact with and navigate within the virtual environment on-screen [4].

A study of 10 stroke patients found that, by wearing a head-mounted display, immersive VR mirror therapy could treat poststroke upper limb paresis [5]. Studies have also been done using VR training to improve lower limb function following a stoke; however, these studies are more infrequent [6]. Immersive VR appears to have greater effects on patient recovery compared to nonimmersive VR [7, 8]. Another study showed that providing patients with unilateral or bilateral limb mirror exercises in a fully immersive virtual environment can activate mirror neurons in damaged areas of the brain, enhance cortical reorganization, and improve motor function [9]. However, other reports have noted there is no obvious correlation between the level of immersion and hemiplegia recovery [10].

Some studies have suggested that VR has more significant effects than conventional therapy in improvement of gait speed, stride frequency, and step length [11], and can improve dynamic balance control, which could prevent patients from falling [12]. However, a study reported that VR and interactive video games are not better than traditional therapies in improving upper limb function [13]. Thus, a nuanced answer in the long-standing debate of the superiority of VR or conventional therapy is that the concept of VR is too general and broad. For example, some methods of VR are simply to improve the fun of rehabilitative exercise to promote patient adherence. In general, head-to-head comparisons between VR and conventional therapy can only be made when VR incorporates the principles of neurorehabilitation [14], which should be the subject of future research efforts.

Though promising, VR technology does have room for further development as VR can induce eye fatigue and physical fatigue during treatment, commonly manifesting as motion sickness. Motion sickness occurs when the screen display and the user's visual response are delayed [15]. Some studies claim, within the virtual environment, patients with hemiplegia use less wrist extension and more elbow extension at the end of the placement phase during arrival, grasping, and performing VR tasks [15]. VR using head-mounted displays is also slower than that in the real environment, and the spatiotemporal kinematics between VR and the real environment is also different [16]. The advantages and disadvantages of VR are summarized in Table 1.

### 2.2. Robot Training (RT)

Rehabilitation robotics have expanded in recent years as the result of extensive communication and interaction between clinicians and engineers, leading to the development of new technologies that stimulate [17]. Rehabilitation robots are different from traditional rehabilitation equipment, as they have a logical control system that can automatically complete a series of complex operations to aid in rehabilitation treatment [18]. Robot systems in the rehabilitation field include exoskeleton and end-effector type robots [19]. Advanced robotic systems can also provide highly repetitive, reproducible, and interactive training forms. It is also possible to use technology developed to evaluate sports performance objectively (e.g., biomechanical data such as speed and strength) in the analysis and evaluation of stroke patient recovery [20].

One study included patients with subacute stroke that were given three weeks of intensive robot training. At the conclusion of the study, their athletic and living ability significantly improved [21]. Another reports that chronic stroke hemiplegia patients receiving three months of robotic training had improvements in proprioceptive control, reactive balance, and posture control [22]. However, a systematic review and meta-analysis found that although RT has the benefit of low labor cost, when the same amount of RT is employed as conventional treatment, RT alone is not better than conventional treatment [23]. Is there a bias in efficiency studies because of one type of robot? A systematic review and meta-analysis study comparing six types of 28 different robotic devices showed that no one type of robotic device is better or worse than other robotic devices [24]. Thus, while current research shows that there is a significant improvement before and after RT treatment, there is no obvious advantage compared with the same amount of conventional treatment and when RT is used as an auxiliary method of conventional treatment, the clinical treatment effect is strongest when the two are combined.

The current paradigm is that many repetitions of rehabilitation actions are effective for promoting recovery. However, traditional rehabilitation therapists have limited energy and the number of repetitions they can manually perform is too small to induce neuroplasticity. Thus, robot-based rehabilitation training may lead to higher exercise repetition intensity, which could promote neuroplasticity. However, some robots, such as Robot-Assisted Step Training (RAST) [25], provide active intervention that does not allow for movement errors or allow patients to take corrective measures. This training can only involve mechanical repetitive training actions, which often reduces the patient's initiative to participate, due to lack of engagement. Thus, researchers have put forward the concept of “assist-as-needed” [25] which refers to helping patients with rehabilitation exercises with the least assistance, so as not to reduce patient spontaneity and initiative. In contrast to this model, a single-blind randomized controlled trial study showed that passive intervention robots are more effective in the rehabilitation of patients with hemiplegia after stroke, and the cost and complexity of passive intervention robots are lower than those of active intervention robots [26].

Some recent research has also studied the portability and comfort of robots. For example, a biofeedback wearable robot based on human-computer interaction, compared to EMG feedback, can better improve patient compliance and can help accelerate the recovery of ankle-foot deformity after stroke [27]. Another study of wearable robots showed that the step symmetry of all stroke patients was significantly improved after training [28]. ReWalk ReStore™ is a soft robotic exosuit that is designed to assist stroke patients in walking through actively assisting the ankle joint [29]. The data recorded by wearable sensors can be used to build models and reduce the high cost and time-consuming efforts of RCT verification. This may lead to the development of robots for specific types of patients in a faster and more targeted manner [30]. The advantages and disadvantages of RT are summarized in Table 2.

### 2.3. mHealth

Rehabilitation of stroke hemiplegia is a long process, and the rehabilitation clinic resources are limited. When discharged from the hospital, hemiplegic patients are usually provided with a written family exercise plan to guide their recovery in the chronic stage of stroke. However, these plans rely on the patient's consciousness, and are unsupervised, which can limit efficacy. To address this, mobile health (mHealth) can provide remote monitoring and remote consultation [31]. mHealth can also provide people living in remote and impoverished areas with access to equitable rehabilitation services [32].

One mHealth example is smart shoe technology based on the Personalized Self-Management Rehabilitation System (PSMrS), which monitors the patient's movement through inductive insoles and projects sensor data on screen to provide feedback to the patient that can be assessed and monitored at home [33]. Another technology is mRehab, a mobile medical technology that combines smartphones and 3D printing and can better support stroke patients with hemiplegia attempting upper limb rehabilitation programs at home [34]. There have also been reports of new technologies that combine egocentric cameras and computer vision algorithms to allow stroke patients with hemiplegia to measure and evaluate hand function at home [35]. Other examples of mHealth include real-time sensor data combined with decision-making algorithms. For example, 70% of users of a music-based digital therapy instrument that helps perform a personalized rhythmic exercise training program to train walking speed after stroke said that they use the device at home most of the time [36]. Similar devices also exist that continuously acquire data without interference so that the patient's motor function can be evaluated in daily life [37]. The ability for patients to perform exercise assessments without a therapist makes assessments more convenient for patients and reduces the cost of medical treatment [38]. The advantages, disadvantages, and technical requirements of mHealth are summarized in Table 3.

## 3. Brain-Computer Interface (BCI)

Brain-computer interface (BCI) is a direct connection path established between the human brain and external devices. This technology translates the neurophysiological signals in the brain into control signals that can operate external devices or computers to assist in performing different tasks [39]. The accuracy of the cortical signals obtained by noninvasive BCI systems is not as high as the signals from invasive BCI [40], but portability, safety, comfort, and low cost make noninvasive BCI the first choice for obtaining relevant brain electrical signals and electroencephalogram (EGG) [41]. These devices include wireless EEG which offers reduced noise and signal artifacts that can be generated by the movement of wired EEG devices [42].

Many clinical studies have shown that BCI training is effective in the rehabilitation of hemiplegia after stroke [43–45]. The combination of BCI and functional electrical stimulation (FES) can also lead to superior clinical outcomes, as FES can enhance the patient's motor awareness and corticospinal excitability during exercise training, which enhances the closure of the sensorimotor circuit in BCI training [46]. Other studies have also found that the combination of BCI and tDCS training in chronic stroke patients enhances the integrity of white matter structures in the brain, increases excitability of the cortex on the same side of the lesion, and improves cerebral blood flow of the parietal and occipital lobes [47]. Despite significant short-term improvement of upper limb motor function after stroke, BCI has not been shown to produce long-term effects. SMR, the target of EEG, has been recently shown to have great potential for improving patients' exercise ability through neurofeedback procedures based on SMR-BCI. In a study that used 20 sessions of SMR-BCI neurofeedback training, patients showed significant upper limb motor recovery which was observable on functional magnetic resonance imaging (fMRI) as an increase in hemisphere activation on the ipsilateral side of the stroke lesion [48]. Quantitative electroencephalogram can also be used for clinical prognosis and monitoring after stroke in acute/subacute stages and can provide a reference value during chronic recovery stages of rehabilitation [49].

BCI via wearable and wireless EEG headsets can record EEG signals in different environments, making EEG-BCI more flexible, yet the recording quality of current headset technology usually declines after about an hour [50, 51]. Dry EEG sensors have also been developed to replace traditional wet sensors and do not require humidifying electrodes or applying gel on the skin. Dry electrodes have technical limitations however, in that they can cause significant scalp discomfort and are very sensitive to muscle and movement artifacts [52, 53]. BCI-based forehead EEGs have also been developed to assess sleep quality and can also be used as a depression treatment screening system, providing new possibilities for the treatment and evaluation of stroke patient sleep and depression risk [54]. Future efforts to improve BCI for neurorehabilitation include the development of “flexible electronics” [55] that can provide a flexible hardware platform for signal amplification to achieve closed-loop interaction, in addition to precise sensing functions. The advantages and disadvantages of BCI are summarized in Table 4.

## 4. Noninvasive Brain Stimulation (NIBS)

### 4.1. Transcranial Direct Current Stimulation (tDCS)

Transcranial direct current stimulation (tDCS) is a form of noninvasive brain stimulation (NIBS). Under physiological conditions, competition between cerebral hemispheres maintains the balance of bilateral cortical excitability. After a stroke, the balance between the hemispheres is disrupted, the excitability of the affected hemisphere decreases, and the excitability of the unaffected hemisphere increases [56]. In tDCS, an anode electrode (+) is usually placed on the affected brain area to increase excitability, whereas the cathode electrode (−) is placed on an unaffected brain area to inhibit excitability [57]. tDCS regulates the resting membrane potential and changes the spontaneous discharge rate through the use of low-amplitude direct currents applied by sponge surface electrodes soaked in salt water [58] to induce neuroplasticity [59].

tDCS is widely used clinically and has significant effects on the recovery of gait speed and gait quality [60]. Studies have found that tDCS can significantly improve the upper limb function of chronic stroke patients and can have significant effects on the lower limbs in patients with subacute stroke [61]. There are three main clinical applications of tDCS which include anode (+) stimulation, cathode (−) stimulation, and bipolar (+) (−) simultaneous stimulation. A network meta-analysis involving 754 stroke patients with hemiplegia found that cathodic (−) tDCS can improve the activities of daily living in patients with hemiplegia after stroke [62]. Preclinical animal studies have also shown that the limb strength and gait of animals treated with cathodic (−) tDCS lead to complete recovery, but animals treated with the anode (+) tDCS only recovered their gait and not limb strength [63]. Cathodic (−) tDCS has also been shown to reduce edema, inflammation, cell apoptosis, and cortical glutamate, creatine, and taurine levels. Cathodic (−) tDCS also preserves cell structure within the cerebral cortex which can lead to reduction in infarct volume and better recovery of function [64]. Other theories of cerebral interactions during the recovery from exercise suggest that the cerebral hemispheres work in cooperation rather than competition. Compared to unipolar stimulation, bipolar stimulation (+) (−) can produce greater performance improvement [65] and future research should focus on bipolar simultaneous stimulation.

The study found that stimulation showed more significant changes in interval stimulation after stroke (such as day 3, day 7, and day 14) compared to daily tDCS. The results showed that the density of cortical dendritic spines increased significantly and the expression of pannexin-1 mRNA involved in hypoxia depolarization decreased [66]. Current density is the main determinant of the efficacy of tDCS, and it is generally believed that the greater the current density is, the better the effect will be, which can activate neurotrophic factors and increase calcium current [67]. However, recent studies have shown that, in anodic tDCS, the excitability change caused by 0.013 mA/cm2 current density is significantly greater than that caused by 0.029 mA/cm2 current density and is sufficient to activate calcium channels and increase intracellular calcium content. Appropriate current density is important because the higher the current density, the deeper the penetrated electric field, which is likely to affect the excitability of undamaged neurons. If the electrode size is too large, it will not only stimulate the target area, but also affect the adjacent cortex [59]. Participants in another trial reported that they had discomfort such as itching and burning during treatment [68]. Traditionally, tDCS uses two common large electrodes for treatment; however, the use of multiple small electrodes may help optimize the applied current, thereby achieving effective targeted stimulation while ensuring the safety of stimulation. In this way, personalized stimulation therapy can be customized for different patients [69]. The advantages and disadvantages of tDCS are summarized in Table 5.

### 4.2. Repeated Transcranial Magnetic Stimulation (rTMS)

rTMS is another type of noninvasive brain stimulation (NIBS), which modulates the excitability of neurons by passing current through an insulated coil. The adjustment of excitability depends on the rTMS parameters. High-frequency stimulation can have excitatory effects within the stroke-damaged hemisphere, and low-frequency stimulation with inhibitory effects is used for the undamaged hemisphere [70]. High-frequency rTMS acts on the brain tissue through a pulsed magnetic field that promotes nerve cell depolarization and can stimulate the neurons of the cerebral cortex to speed up the reconstruction of neural pathways, thereby improving nerve function [71]. Low-frequency rTMS uses pulsed magnetic fields to activate inhibitory circuits in the cortex to inhibit brain nerve activity [72].

rTMS can delay or prevent the death of hippocampal neurons in adult rats with cerebral ischemia. The specific preservation of neurons depends on the stimulation mode and the time interval between ischemia and stimulation. Maximum neuronal protection can be achieved by applying high-frequency rTMS (at least 128 seconds) in the first 48 hours of ischemia occurring [73]. Ischemic lesions can also cause a decrease in the expression of microtubule-associated protein 2 and mitochondrial axon transport, which leads to a decrease in ATP utilization and, ultimately, neuronal death. In rats treated with high-frequency rTMS, the expression of microtubule-associated protein 2 and ATP content in the diseased hemisphere increased significantly, suggesting that neuron repair was in progress [74]. Moreover, the expression of c-Fos and brain-derived neurotrophic factor in the cortex of rats treated with rTMS also increased significantly and promoted stroke recovery [75]. Other studies have shown that the application of 10 Hz rTMS treatment to the diseased hemisphere for 7 days significantly increases the proliferation of adult neural stem cells in the ventricle on the side of the lesion [76].

Although rTMS has been widely used to improve upper limb movement in stroke patients with hemiplegia, a systematic review and meta-analysis of 199 patients showed that current literature is insufficient to support the conclusion that rTMS combined with upper limb training is more effective than upper limb training alone [77]. The 2009 rTMS Clinical Guidelines indicate that 10 courses of rTMS are optimal; however, a 2017 study found that 5 courses of rTMS treatment are best for improving stroke-induced dyspraxia. This study also found that more than 5 courses of rTMS treatment did not have a better effect on the recovery of motor function, especially after more than 10 courses of treatment, in which case the therapeutic effect of rTMS actually decreased [78].

Theta burst stimulation (TBS) is a newly developed form of rTMS that mimics the firing patterns of hippocampal pyramidal cells during wakefulness in rodents exposed to new environments, producing low-intensity bursts of stimulation to coordinate cortical excitability [79]. Intermittent theta burst stimulation (iTBS) has been shown to enhance cortical excitability, while continuous theta burst stimulation (cTBS) inhibits cortical excitability [80]. iTBS lasting for 10 days can also promote the development of nerves in the ipsilateral inferior ventricle and increase outgrowth of nerve progenitor cells [81], in addition to enhancing neuron excitability and improving motor ability. Although cTBS inhibits excitability in the unaffected hemisphere, it does not improve hand motor function. Thus, iTBS appears to be more beneficial for patients' limb recovery than cTBS. In addition, TBS increases the risk of epileptic seizures [78]. The advantages and disadvantages of rTMS are summarized in Table 6.

## 5. Traditional Chinese Medicine Rehabilitation and Interdisciplinary Combination of Medicine and Engineering

Traditional Chinese medicine (TCM) is a treasure of the Chinese nation and when combined with modern science and technology can result in truly optimal integrations of medicine and engineering to advance TCM rehabilitation technology. TCM treatment of stroke hemiplegia typically includes acupuncture, massage, and rehabilitation training, supplemented by drug, psychological, physical, and exercise therapy, to promote the recovery of limb function. TCM first began taking advantage of advances in medicine and engineering through combination of acupuncture and electricity in the 1950s when Zhu Longyu established electroacupuncture therapy [82]. Acupuncture can improve the excitability of residual nerve cells, promote neuroplasticity in the damaged area, and reduce muscle tension. Electroacupuncture is mainly used to stimulate muscle movement by infusing low-frequency current stimulation on the skeletal muscle through needles, to achieve the purpose of enhancing the effect of acupuncture [83]. Electroacupuncture is currently the most widely used TCM for the treatment of poststroke hemiplegia. Newer technologies that also incorporate TCM include combinations of robotics, ultrashort wave, semiconductor lasers, ultrasound, and hyperbaric oxygen. Zhang [84] used acupuncture and a Lokohelp robot to treat patients with acute ischemic stroke hemiplegia, which significantly improved the patient's neurological deficits and improved the patient's walking ability, balance function, motor function, and activities of daily living. Ultrashort wave therapy uses the microthermal effect of electromagnetic fields to not eliminate only inflammatory cells, while simultaneously promoting edema absorption and accelerate microcirculation. Ultrashort wave therapy can also reduce sympathetic nerve tension, reduce vasospasm, establish collateral circulation, and nourish nerve tissue [85]. Li and Lai [86] showed that rehabilitation training combined with ultrashort wave therapy had significant effects in patients with hemiplegia after stroke stage I shoulder-hand syndrome. The mechanism of the semiconductor lasers is similar to that of the ultrashort wave, except that it uses light energy to reduce swelling, inflammation, and analgesia. Zhu [87] has achieved good clinical effects by using a semiconductor laser with electroacupuncture and rehabilitation training. Ultrasound uses waves that can produce physical, chemical, thermal, and mechanical effects, as well as cause tissue cytoplasm to flow and rotate. This can change the PH value in the tissue, improve the permeability of the biofilm, and accelerate the blood circulation and metabolism of the tissue [88]. Xu et al. [89] have also achieved excellent results in the treatment of hemiplegic shoulder pain after stroke using ultrasound introduction combined with rehabilitation training. Acupuncture combined with hyperbaric oxygen can have a synergistic effect in improving the oxygen supply status to the brain. This method can improve brain tissue energy metabolism, restoring the aerobic metabolism of nerve cells in the ischemic penumbra area, and scavenging oxygen free radicals. Hao [90] used Xingnao Kaiqiao acupuncture combined with hyperbaric oxygen to treat stroke hemiplegia and achieved significant clinical effects. New techniques combining sound, light, and electricity also serve as comprehensive treatments, which aid in recovery of peripheral and central nerves, effectively avoiding the limitations in space and time of conventional treatment with three kinds of equipment [91]. The advantages and disadvantages of TCM rehabilitation are summarized in Table 7.

## 6. Discussion

With the rapid development of science and technology, artificial intelligence, brain-computer interface, and other technologies have been widely used in clinical practice. Interdisciplinary combination of medicine and engineering is an inevitable trend in the development of modern medicine. However, as summarized above, there are some areas to be improved regarding appropriate patient selection and technical optimization. A major problem that exists is that most of the current interdisciplinary combination of medicine and engineering methods cannot prove that they are better than conventional therapy when used alone, and require further technical optimization. Additionally, in some studies, patients in the subacute phase after stroke were included in the comparison of treatment methods, but these data were not convincing because spontaneous recovery in the subacute phase would interfere with experimental results. There are also unique differences among stroke patients with hemiplegia, such as those suffering from ischemic or hemorrhagic stroke or those in the subacute or recovery phases. A limitation of some studies is that they do not specify the category of patients in the study. Many interdisciplinary combinations of medicine and engineering treatment methods also lack standardized programs for intervention measures, stimulation parameters, and treatment course standards, which likely contribute to a lack of mechanistic insights regarding these methods. The interdisciplinary combination of medicine and engineering in the field of TCM rehabilitation has great potential, but the foundation is relatively weak, and further development and research are needed.

In addition to helping doctors treat patients and promote their recovery, interdisciplinary combination of medicine and engineering has also been initially used to predict the incidence and prognosis of stroke hemiplegia. Chen and Song [93] established a stroke recurrence prediction model based on big data to assess the risk of stroke recurrence and achieved a prediction accuracy of 83%. This study found that the top 9 factors affecting recurrence are age, hypertension, triglyceride, coronary heart disease, family history of hypertension, body mass index, total cholesterol, homocysteine, and high-density lipoprotein. Liang et al. [94] also used big data to establish a stroke platform based on the new model of “Internet Plus Disabled Community Rehabilitation,” providing a standardized model for describing the rehabilitation of stroke patients and at the same time, providing a platform for effective information exchange regarding rehabilitation institutions. One of the most dangerous complications of long-term oral anticoagulant therapy (OAT) is associated with intracerebral hemorrhage (OAT-ICH). The allele *ε*2/*ε*4 of apolipoprotein E (APOE) is strongly associated with recurrence of OAT-ICH. Biffi et al. [95] used neuroimaging to detect MRI markers of APOE *ε*2/*ε*4 variants to predict OAT-ICH recurrence. Liew et al. [96] also used the combination of neuroimaging and big data to establish the ENIGMA Stroke Recovery Working Group to predict the recovery of stroke patients.

Interdisciplinary combination of medicine and engineering refers to integration and collaborative innovation of medicine and engineering centering on existing medical needs. This allows the most advanced technological means of engineering to help solve clinical needs, and aid doctors in quick and accurate diagnoses, which promotes rapid patient recovery [92]. However, there are still many bottlenecks in the development of optimal interdisciplinary combination of medicine and engineering. For example, substantial integration is not widespread and current efforts are primarily concentrated within specific disciplines, producing many “one-to-one” and not “one-to-many” or “many-to-many” interdisciplinary models. There is also a critical lack of highly educated talents with medical and engineering backgrounds. Science and engineering students do not sufficiently understand clinical medicine and medical students do not have relevant knowledge of science and engineering, which leads to the current situation of knowledge separation and difficult integration. The industry-university-research chain is also not perfect as hospitals, enterprises, and schools at times lack effective communication and separate projects that are not openly discussed limit the ability for widespread research advancement. Insufficient policy and financial support have also limited interdisciplinary combination of medicine and engineering.

To break through the bottleneck of development, in addition to the special support of policies and funds, it is suggested to promote the interdisciplinary research of medicine and biology, physics, material science, computer science, and other disciplines, to form many cross-disciplines with the characteristics of interdisciplinary combination of medicine and engineering. However, the most important thing for cultivating high-level interdisciplinary combination of medicine and engineering talent is the promotion cross-disciplinary thinking and innovation for trainees who are proficient in medical and engineering knowledge and can actively find problems encountered in clinical practice and solve them with engineering methods.

## Figures and Tables

**Table 1:** Virtual reality advantages and disadvantages.

Virtual reality	
Advantages	Low labor cost, high safety, improved training fun, and increased patient adherence [3, 4]

Disadvantages	(1) Vertigo [15]
	(2) Varying head-mounted display weight and comfort [15]
	(3) Eye fatigue [15]
	(4) Difference in the efficacy of immersion vs. nonimmersion therapy [10]
	(5) Limited research on efficacy in lower limbs [13]
	(6) Research on VR with neurorehabilitation principles should be increased [14]

**Table 2:** Robot therapy advantages and disadvantages.

Robotic therapy	
Advantages	Low labor cost, no trauma, no side effects, simple operation, and high conversion rate of economic benefits [18–20]

Disadvantages	(1) Generally large in size, poor in portability, and lack flexibility [27, 28]
	(2) Low comfort [29]
	(3) Personalized RT plans are needed for specific populations (e.g., subacute and chronic phases) [30]
	(4) Robot interfaces can be boring and fatigue-prone, necessitating development of a more friendly human-machine interface and with interesting games [30]
	(5) Robot mechanical structure and control systems lack real-time and precise control of the angle and speed of the patient's joints [25]
	(6) Not optimized for slower responding elderly patients [30]

**Table 3:** Mobile health advantages, disadvantages, and technical requirements.

Mobile health	
Advantages	Improve the efficiency of patients' self-rehabilitation exercises, provide services to people in remote areas, and reduce medical costs [31]

Disadvantages	(1) Sampling at a low rate may cause information loss [37]
	(2) Noise during exercise can affect data sampling [37]
	(3) Problems with the battery life of the device [38]
	(4) Regular maintenance and cleaning issues [38]
	(5) Putting on and taking off devices when used on disabled patients [34]

Technical requirements	(1) The patient can operate as much independently as possible [38]
	(2) Technical safety must be guaranteed [38]

**Table 4:** Brain-computer interface advantages and disadvantages.

Brain-computer interface	
Advantages	Invasive BCI: high accuracy [40]
	Noninvasive BCI: portability, safety, comfort, and low cost [41]

Disadvantages	(1) Side effects: short-term nausea, fatigue, and headache [43–45]
	(2) Small studies, limited research [55]
	(3) No long-term effects [48]
	(4) Lack of comparison between BCI and traditional therapies [48]
	(5) Unknown which stage of poststroke hemiplegia is best for BCI training [49]
	(6) Limited research on efficacy in lower limbs [43–45]

**Table 5:** Transcranial direct current stimulation advantages and disadvantages.

Transcranial direct current stimulation	
Advantages	Relatively cheap, easy to manage, and carry [56–58]

Disadvantages	(1) Side effects such as pain, itching, and burning sensation [68]
	(2) Optimal current density is unknown [59]
	(3) Optimal stimulation parameters (anode/cathode/bipolar) are unknown [62–65]
	(4) Optimal treatment duration is unknown [66]
	(5) Future research should focus on designing more personalized tDCS stimulation programs for patients [69]

**Table 6:** Repeated transcranial magnetic stimulation advantages and disadvantages.

Repeated transcranial magnetic stimulation	
Advantages	Relatively cheap, easy to manage and carry [70–72]

Disadvantages	(1) The effect of rTMS in different periods poststroke is unknown [73]
	(2) Lack of standardization with unknown optimal course of treatment [78]
	(3) The optimal stimulation parameters of rTMS need to be further determined [76]
	(4) At present, a lot of evidence does not support the individual efficacy of rTMS, and rTMS technology needs to be further optimized [77]

**Table 7:** The advantages and disadvantages of interdisciplinary combination of medicine and engineering in TCM rehabilitation.

Traditional Chinese medicine rehabilitation and interdisciplinary combination of medicine and engineering	
Advantages	Combination therapy has better clinical effects than single therapy [84–91]

Disadvantages	(1) Late start, weak foundation, and lack of hardware measures [92]
	(2) Lack of high-innovation teams and compound leading talents [92]
	(3) Lack of quantification, standardization, and standardization [92]

## Data Availability

No data were used to support this study as this is a review article.
